# Supporting primary health care led preventive screening through community-embedded health access points in Ukraine

**DOI:** 10.3389/fpubh.2026.1834596

**Published:** 2026-05-29

**Authors:** Halyna Darahan, Roman Rodyna, Myroslava Germanovych, Aleksey Bogdanov, Natalia Zaika, Kateryna Gamazina, Eveline Klinkenberg, Gunta Dravniece

**Affiliations:** 1 PATH, Kyiv, Ukraine; 2 ConnectTB, The Hague, Netherlands

**Keywords:** community-embedded, health access point, noncommunicable disease, primary healthcare, Ukraine

## Abstract

**Introduction:**

Ensuring timely, affordable access to preventive screening and early detection of noncommunicable diseases (NCDs) is essential for resilient primary health care (PHC), yet coverage remains uneven in rural and hard-to-reach communities in Ukraine. Long-standing barriers related to distance, transport and limited local services have been made worse since 2022 by a full-scale war. To operationalize the national policy and strengthen local health services access, the Support TB Control Efforts in Ukraine project supported Health Access Points (HAPs) using a community-embedded model to decentralize screening and reinforce referral pathways to PHC.

**Methods:**

HAPs were operationalized in medical facilities, and at community venues, i.e., libraries, employment offices, social service centers. Screening offered to all adults included blood pressure, glucose, cholesterol, cancer risk assessment (breast, cervical, prostate, colorectal), immunization status, and depression. Health workers collected data using standardized registers and aggregated it monthly for descriptive analysis.

**Results:**

Between December 2023 and June 2025, 234 HAPs were operationalized across 13 hromadas in 11 oblasts. A total of 92,018 screening records were included, 75.8% from primary visits and 24.2% from secondary visits. Most attendees (83.2%) were aged 40 years or older, while 16.8% were aged 18–39 years, demonstrating uptake beyond the nationally prioritized 40 years and older age group. Nearly all attendees (98.0%) had their blood pressure taken, and 7.7% (95% CI: 7.5–7.9%) of these had hypertension. Among people who received depression screening, 7.2% (95% CI: 7.0–7.4%) were referred for further evaluation. Cancer risk assessments indicated substantial needs for referral: 21.8% after breast cancer screening, 24.1% after cervical cancer screening, and 23.2% after prostate cancer screening. Immunization checks identified gaps requiring referral for a tetanus and diphtheria booster for 15.1% (95% CI: 14.9–15.4%) of those screened.

**Conclusion:**

The HAP model demonstrated a feasible and scalable approach to expand preventive screening as an extension of PHC with referral closer to communities during wartime, including for adults younger than 40 years. Embedding services in community venues provide people and especially internally displaced persons with impactful, time-effective, gender-sensitive and age-responsive integrated quality health services, for communicable and noncommunicable diseases as well as strengthen the prevention services in conflict-affected settings.

## 1 Introduction

Universal access to timely, affordable, and high-quality health services is a cornerstone of resilient and equitable health systems (1). Preventive screening for noncommunicable diseases (NCDs) and mental health conditions is central to achieving this vision, enabling early detection and referral to reduce the burden of preventable disease (2) especially in crisis situations (3, 4).

NCDs—including cardiovascular diseases, diabetes, chronic respiratory conditions, and cancers—represent the leading cause of death and disability in Ukraine, accounting for approximately 73% of all mortality in 2021 (5–7). Ukraine has one of the highest mortality rates from cardiovascular diseases in Europe, with 772.1 deaths per 100,000 males and 440.9 per 100,000 females in 2017 (8). In 2019, almost 30% of men who died from NCDs were under 60 years of age (9). Key contributing factors include tobacco use, unhealthy diets, physical inactivity, and gaps in preventive and primary care coverage (8–10). Access to systematic screening and preventive interventions has historically been limited in rural and remote areas, resulting in delayed diagnoses and poorer outcomes (5, 11, 12).

The full-scale war that began in 2022 further disrupted primary health care (PHC) delivery. Damage to infrastructure, population displacement, workforce shortages, and disrupted supply chains compounded preexisting inequities. Vulnerable groups—including internally displaced persons (IDPs), people with chronic conditions, and residents of recently de-occupied or hard-to-reach communities (hromadas)—face particularly high barriers to care (13, 14).

At the policy level, NCD screening in Ukraine is regulated by Ministerial Orders. Order No. 504 (March 19, 2018) established a framework for preventive screening protocols aimed at early detection and management of common NCDs in PHC settings (15). The order outlined screening recommendations, including target populations, screening methods, and suggested frequency for different conditions. A subsequent update, Order No. 1834 (November 1, 2024), reinforced and expanded these recommendations by emphasizing screening in PHC, better patient access to diagnostic services, and the need for strengthening training programs for PHC personnel (16). It also highlighted the importance of colorectal cancer screening as a critical intervention due to its high morbidity and mortality in Ukraine (16).

Despite these policies, uptake of preventive services remained suboptimal, especially in rural and conflict-affected areas (11, 13). To address these gaps, the Support TB Control Efforts in Ukraine (STBCEU) project, funded by the U.S. Government, and implemented by PATH, together with national and regional health authorities introduced a model of HAPs. HAPs were operationalized as community-embedded hubs aiming to bring services closer to residents by decentralizing screening for NCDs and mental health care and strengthening referral pathways to PHC.

During the study period and at the time of manuscript revision, Ukraine further updated its regulatory framework for preventive screening, including Cabinet of Ministers Resolution No. 1652 (2025) and Ministry of Health Order No. 19 (2026), which reinforce the organization of screening services within primary health care. The HAP model aligns with these updated regulations by functioning as an outreach and demand-generation mechanism linked to PHC-based diagnostic and treatment services.

## 2 Methods

### 2.1 HAP establishment

HAP implementation followed a stepwise approach. In early 2023, online meetings were conducted with oblast health departments and regional Centers for Disease Control (rCDCs) in five regions (Chernihiv, Kyiv, Rivne, Zhytomyr, and Vinnitsa). During these online meetings, the plans to strengthen NCD screening at the community level in response to health system challenges caused by the war (restricted access to medical services in de-occupied areas, PHC overload in communities with large numbers of IDPs) were further developed, including operationalization of the HAP system. Regions were asked to identify and propose one hromada per region for HAP implementation, either in de-occupied territories or in hromadas with high numbers of IDPs. Subsequently, assessment visits were conducted to each identified hromada to assess the NCD burden and current NCD screening practices. At the same time, initial meetings were held with senior management of hromadas, representatives of oblast health departments, and rCDCs to jointly agree on the proposed approach for HAP implementation, as well as what type of support would be most helpful from the STBCEU project.

The assessments revealed a high burden of NCDs, including hypertension and diabetes, and significant service access barriers, including health care worker shortages, limited transportation infrastructure, and internal displacement due to conflict. Together with local hromada leadership, the project developed selection criteria for HAP placement based on local health system vulnerabilities, population access barriers, and the presence of IDPs. In the selected hromadas, planning meetings were held to establish roles and responsibilities, select locations for HAPs using the set criteria, and ensure that services offered would align with national health guidelines and support existing practices. This participatory planning process ensured complementarity with local systems and policy frameworks and supported local ownership and sustainability.

HAPs were operationalized in both medical and community sites in alignment with national health protocols. Medical sites included rural outpatient clinics and village health posts, staffed by nurses or family doctors. Community sites included libraries, cultural clubs, hromada offices, employment centers, social protection offices, educational facilities, and local markets. These sites were operated by trained community actors—such as librarians, social workers, volunteers, and in some hromadas medical staff. HAP staff carried out NCD screening, provided health education, and facilitated referrals to PHC where needed. HAPs functioned as community-level screening and referral entry points. In contrast, PHC facilities were responsible for clinical evaluation, diagnostic confirmation, and treatment initiation. HAP operation commenced in a phased approach with different regions starting at different timepoints to allow for gradual expansion of project activities and to incorporate the learnings from HAP operationalization in other hromadas.

### 2.2 Screening strategy and approach

Per national guidelines, NCD screening is mandated for all individuals aged 40 years and above. In addition, for women 21 years and older, breast and cervical cancer screening is recommended, while for men 40 years and older, prostate cancer screening is recommended. In recognition of war-related stress and its potential impact on the population, the project expanded the screening scope by providing NCD and cancer screening for all individuals 18 years and older regardless of risk factors.

The project created awareness among the target population through outreach and community engagement, followed by sensitization and invitation for screening through information campaigns using posters, public announcements, and word of mouth. At a HAP, all attendees received brief counseling and health education materials before the NCD screening. Each HAP was equipped with automatic blood pressure (BP) monitors, glucometers, cholesterol meters (with strips and reagents), digital scales, and stadiometers, and operated by trained personnel who received standardized instruction on screening protocols, use of equipment, identification of risk factors, and referral procedures. Educational materials on NCD prevention, immunization, and mental health were displayed at each venue and distributed to attendees. Measurement for blood pressure and glucose, colorectal cancer screening, status of preventive immunization, and mental health screening was offered to all attendees. Blood pressure ≥140/90 mm Hg indicated hypertension, and glucose thresholds ≥5.5 mmol/L (fasting) and ≥7.8 mmol/L (non-fasting) were considered deviations from the norm and warranted referral for further evaluation. Colorectal cancer screening consisted of a questionnaire about risk factors and symptoms. Preventive immunization status was evaluated by inquiring whether attendees had received immunization against diphtheria and tetanus within the last 10 years. Attendees who reported not having received these were referred to the PHC level for a tetanus and diphtheria (Td) booster vaccination. Mental health screening was done using the PHQ-9 questionnaire, a self-administered tool designed to screen for and measure the severity of depressive symptoms (17). Individuals scoring ≥10 on the PHQ-9 were referred to PHC for further evaluation. Furthermore, all attendees, regardless of their PHQ-9 score, received educational material focusing on stress prevention and alleviation techniques. Screening for prostate cancer was offered to all male attendees and screening for breast and cervical cancer was offered to all female attendees. Individuals with risk factors identified based on cancer screening were referred for further evaluation and diagnostic confirmation, either at the PHC or specialized care level. Table 1 summarizes the screening services by target group, method used and threshold for referral.

**Table 1:** Screening services offered at health access points.

Condition	Target population	Screening tool or method	Referral threshold
Hypertension	Adults 18+	Blood pressure measurement	Systolic ≥140 mm Hg or diastolic ≥90 mm Hg
Diabetes	Adults 18+	Fasting or random glucose	Fasting ≥5.5 mmol/L or non-fasting ≥7.8 mmol/L
Colorectal cancer	Adults 18 + at risk	Risk assessment and symptom screening	Referral if symptoms or risk indicators present
Prostate cancer	Men 18 + at risk	Risk assessment and symptom screening	Referral based on symptoms or family history
Breast/cervical cancer	Women 18 + at risk	Risk assessment and symptom screening	Referral based on screening results
Depression	Adults 18+	PHQ-9 questionnaire	Score of ≥10 on PHQ-9
Immunization status	Adults 18+	Verbal confirmation	No diphtheria/tetanus vaccine in past 10 years

Screening at HAPs was designed as an initial risk assessment and symptom-based screening rather than diagnostic evaluation. The purpose of HAP-level screening was to identify individuals with potential abnormalities or risk factors and refer them to primary health care (PHC) providers for further clinical assessment and diagnostic confirmation in accordance with national guidelines. Screening procedures requiring clinical skills were conducted by trained healthcare personnel or under their supervision. Non-medical personnel conducted health education, administered structured questionnaires, and supported referral navigation. Diagnostic procedures (e.g., laboratory testing, imaging, or specialist consultations) were not performed at HAPs and were conducted only at PHC or higher levels of care following referral.

For reporting purposes, HAP attendance was classified by visit type. A primary visit was defined as an attendee’s first recorded screening encounter at a given HAP site. A secondary visit was defined as any subsequent visit to the same HAP site, including follow-up screening, repeated measurements for monitoring, or screening for additional conditions.

### 2.3 Training and capacity building

Before HAP activities started, a series of trainings was conducted for each community. PHC doctors led general training orientation on NCD prevention, screening, and epidemiology for key community representatives. Training materials consisted of project-developed presentations based on international guidelines and national regulatory documents. Then, before the launch of HAPs in communities, an online training was conducted for HAP staff on screening procedures, data capture, and reporting, followed by in-person training on NCD screening and referral pathways for a mixed audience, including HAP staff, community representatives, family doctors and PHC nurses, and rCDCs, to ensure consistent implementation and preparedness to manage referrals generated through HAP activities. In addition, just before the actual launch of the HAPs, online refresher trainings were conducted on the screening algorithms, risk factor identification, and data reporting. Training ensured that both healthcare and non-medical personnel were competent in their respective roles, with clinical measurements performed by trained healthcare staff or under their supervision, and structured tools (e.g., PHQ-9) administered by trained personnel.

### 2.4 Data collection, management, and analysis

This study is a retrospective descriptive observational analysis of routinely collected programmatic data from HAPs implemented between December 2023 and June 2025. Each HAP collected data offline using a standardized register. Key variables collected were site identifier, attendee’s demographic characteristics, type of service (screening, referral, education session), conditions screened, visit type (primary, secondary, follow-up), and deviation status (new, treatment monitoring). The unit of analysis is the screening record (visit), not the individual person. For each screening condition, a value was entered in the “new” or “treatment monitoring” column when a deviation (i.e., abnormal result) was identified. If no deviation was found, the fields were left blank. “New” meant first-time deviation from the norm, and “treatment monitoring” was used for monitoring of BP and other conditions at subsequent visits. Primary records were completed in real time and maintained by HAP staff, who transmitted data once a month to the coordinator at hromada level. This coordinator aggregated the data for the different HAPs monthly in Excel and transferred the information to the STBCEU project team. Data quality checks were performed by the STBCEU team to ensure data consistency and completeness. Project supervisors visited each hromada after the opening of HAP, completing a total of 13 monitoring visits. These visits included observation of screening processes, review of collected data, and provision of onsite mentorship as needed including refresher trainings.

Data analysis was mostly descriptive, summarizing the characteristics of the study population using proportions for binary and categorical variables and using means and medians (with 25th and 75th quantiles) for continuous variables.

NCD screening coverage was defined as the number of people screened divided by the total number of eligible people. Screening coverage and results were compared by gender, age group (below or above 40 years), and screening point type (medical or community) using chi-square or Fisher exact tests for binary and categorical variables.

The analysis presented in this manuscript is limited to data collected at the HAP level, including screening coverage, identified deviations from normal values, and referral indications. This study did not assess referral completion, diagnostic confirmation, or treatment outcomes. Data on referral completion, diagnostic outcomes, and treatment initiation at PHC or specialist level were not systematically captured across all sites and are therefore not included in this analysis. The analysis focused on screening reach and referral indication and did not evaluate the effectiveness of referral completion or subsequent clinical management.

## 3 Results

### 3.1 HAP expansion and attendees profile

**Figure 1:**
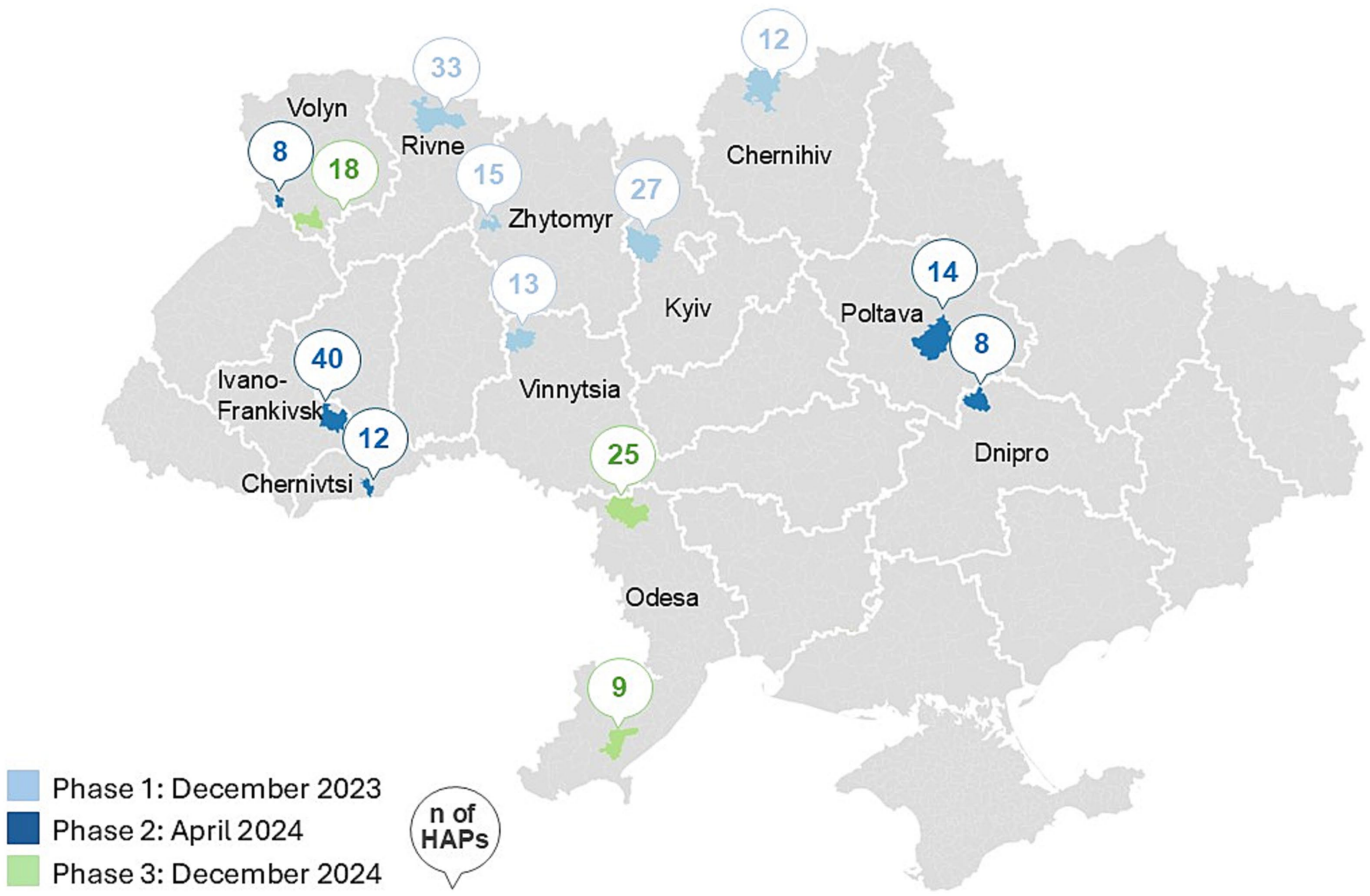
Geographic distribution of hromadas with start date of scale up phase and number of operational health access points from December 2023 to June 2025.

HAP implementation occurred in three phases between December 2023 and December 2024. In December 2023, 100 HAPs in five regions were set up, including 39 HAPs in recently de-occupied areas. Initial sites included Dubrovytska (Rivne region), Zvyahilska (Zhytomyr region), Makarivska (Kyiv region), Horodnyanska (Chernihiv region), and Khmilnytska (Vinnytsia region). In April 2024, the effort expanded to five more regions with 82 HAPs established in Horodenkivska (Ivano-Frankivsk region), Hlybotska (Chernivtsi region), Ivanychivska (Volyn region), Reshetylivska (Poltava region), and Tsarychanska (Dnipropetrovsk region). In December 2024, three more hromadas were added with 52 HAPs established in Baltska and Tatarbunarska (Odesa region) and Gorokhiv (Volyn region). As of June 2025, 234 HAPs were operating in 13 hromadas across 11 oblasts. Of these 234 HAPs, 45 (19.2%) were located in community venues. Figure 1 shows the geographic distribution of the HAPs as well as the three expansion phases.

Across the 234 operational HAPs, from December 2023–June 2025 a total of 95,026 records of separate visits were collated. During data validation, 3,008 (3.2%) records were dropped for missing age, sex not matching the gender-specific screening conducted, or missing type of visit or type of facility, resulting in a total of 92,018 records included in this analysis.

Of the validated records, this was a primary (first) screening visit for 69,733 (75.8%) and a secondary (follow-up) visit for 22,285 (24.2%) as seen in Table 2. Among all attendees, 29,896 (32.5%) were of men and 62,122 (67.5%) of women. Overall mean age was 56.0 years (standard deviation 15.7), and age was similar between men and women (55.3 and 56.4 years, respectively). The majority of attendees seen—76,506 out of 92,018 (83.6%)—were 40 years or older while 15,512 (17.0%) were 18–39 years of age.

**Table 2:** Demographics by visit type for noncommunicable disease screening in 234 health access points across 13 hromadas from December 2024 to June 2025.

# screened	Primary/first visit		Secondary/follow-up visit		Total	
Sex						
Male	24,041	34.5%	5,855	26.3%	29,896	32.5%
Female	45,692	65.5%	16,430	73.7%	62,122	67.5%
Age group						
18–24 years	2,519	3.6%	189	0.8%	2,708	2.9%
25–34 years	6,318	9.1%	748	3.4%	7,066	7.7%
35–39 years	5,010	7.2%	728	3.3%	5,738	6.2%
40–44 years	5,518	7.9%	1,061	4.8%	6,579	7.1%
45–54 years	14,090	20.2%	4,010	18.0%	18,100	19.7%
55–64 years	15,632	22.4%	6,165	27.7%	21,797	23.7%
65 + years	20,646	29.6%	9,384	42.1%	30,030	32.6%
Age category						
18–39 years	13,847	19.9%	1,665	7.5%	15,512	16.9%
40 years and above	55,886	80.1%	20,620	92.5%	76,506	83.1%
Total	69,733	100.0%	22,285	100.0%	92,018	100.0%

Most attendees (84.1% or 77,423/92,018) attended a HAP located at a medical site (Table 3). The proportion of women was significantly higher at HAPs located in community sites compared to medical sites (76.0% versus 65.9%, chi-square 567.9284, *p* < 0.001).

**Table 3:** Characteristics of health access point (HAP) attendance by type of screening point (medical/community).

% (within category)	HAP screening point type					
	Medical		Community		Total	
Sex						
Male	26,391	34.1%	3,505	24.0%	29,896	32.5%
Female	51,032	65.9%	11,090	76.0%	62,122	67.5%
Age group						
18–24 years	2,439	3.2%	269	1.8%	2,708	2.9%
25–34 years	5,950	7.7%	1,116	7.6%	7,066	7.7%
35–39 years	4,680	6.0%	1,058	7.2%	5,738	6.2%
40–44 years	5,423	7.0%	1,156	7.9%	6,579	7.1%
45–54 years	15,046	19.4%	3,054	20.9%	18,100	19.7%
55–64 years	18,067	23.3%	3,730	25.6%	21,797	23.7%
65 + years	25,818	33.3%	4,212	28.9%	30,030	32.6%
Age category						
18–39 years	13,069	16.9%	2,443	16.7%	15,512	16.9%
40 years and above	64,354	83.1%	12,152	83.3%	76,506	83.1%
Total	77,423	84.1%	14,595	15.9%	92,018	100.0%

**Figure 2:**
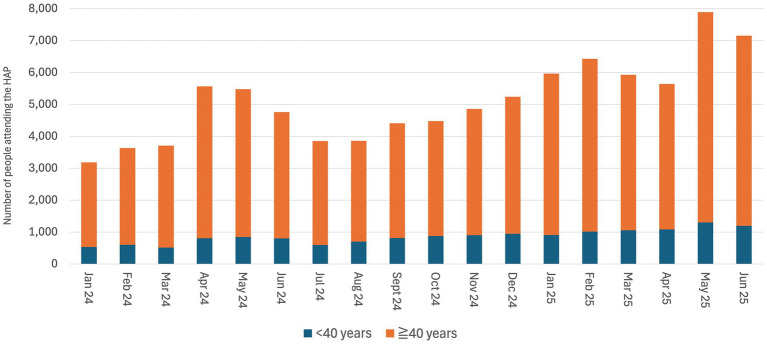
Health access point attendance by month during expansion of services by age group.

Total attendance increased over time, with 3,186 people seen at HAPs in January 2024 and 7,151 by June 2025, though the pattern also fluctuated over time (Figure 2). A significant increase in visits was observed in April–May 2024, coinciding with the opening of 87 HAPs in five new communities. A similar trend occurred between December 2024 and February 2025, linked with the launch of 34 HAPs in Odesa oblast. In summer 2025, an additional increase in visits was linked to the opening of 18 HAPs in Volyn oblast.

### 3.2 Screening outcomes, referrals and follow-up

Screening coverage (the actual number of attendees screened for a condition divided by the number of attendees eligible to be screened for that condition) varied by NCD. The highest coverage was for BP screening at 98.0% and the lowest was for cholesterol at 38.2% (Table 4). Screening coverage was overall similar by gender, except for colorectal cancer for which men were more frequently screened than women (84.2% versus 61.6%, *p* < 0.001). Slightly more men than women had a BP screening result suggesting hypertension (9.0% versus 7.0%, *p* < 0.001) though slightly more women than men underwent treatment monitoring for high BP (25.6% versus 21.6%, *p* < 0.001). Referrals due to deviations from standard cholesterol levels were observed most frequently, occurring in approximately 15% of both male and female attendees. Women had referral rates of 21.8% (95% CI: 21.4–22.2%) after breast cancer screening and 24.1% (95% CI: 23.7–24.5%) after cervical cancer screening. Similarly, a high rate of referral (23.2% (95% CI: 22.7–23.7%)) was observed among men after prostate cancer screening. Depression screening identified 7.2% (95% CI: 7.0–7.4%) of attendees (6.8% men, 7.4% women) with PHQ-9 scores ≥10. Education session on stress management were given to 54.6% of HAP attendees.

**Table 4:** Overview of screening for noncommunicable diseases (NCD) in 234 health access points (HAPs) across 13 hromadas from December 2023 to June 2025 overall, by sex, age group, and type of HAP including referrals made to primary health care (PHC).

	Sex				Age group				HAP screening point type					
	Female		Male		Age <40 years		Age > 40 years		Medical		Non-medical		Total	
Total	62,122	67.5%	29,896	32.5%	15,512	16.9%	76,506	83.1%	77,423	84.1%	14,595	15.9%	92,018	100.0%
Sex														
Male			29,896	100.0%	5,233	33.7%	24,663	32.2%	26,391	34.1%	3,505	24.0%	29,896	32.5%
Female	62,122	100.0%			10,279	66.3%	51,843	67.8%	51,032	65.9%	11,090	76.0%	62,122	67.5%
Age group														
18–24 years	1,684	2.7%	1,024	3.4%					2,439	3.2%	269	1.8%	2,708	2.9%
25–34 years	4,695	7.6%	2,371	7.9%					5,950	7.7%	1,116	7.6%	7,066	7.7%
35–39 years	3,900	6.3%	1,838	6.1%					4,680	6.0%	1,058	7.2%	5,738	6.2%
40–44 years	4,338	7.0%	2,241	7.5%					5,423	7.0%	1,156	7.9%	6,579	7.1%
45–54 years	12,254	19.7%	5,846	19.6%					15,046	19.4%	3,054	20.9%	18,100	19.7%
55–64 years	14,505	23.3%	7,292	24.4%					18,067	23.3%	3,730	25.6%	21,797	23.7%
65 + years	20,746	33.4%	9,284	31.1%					25,818	33.3%	4,212	28.9%	30,030	32.6%

	Sex				Age group				HAP screening point type					
NCD screening	Female		Male		Age 18–39 yrs		Age 40 + yrs		Medical		Non-medical		Total	
Total	62,122	67.5%	29,896	32.5%	15,512	16.9%	76,506	83.1%	77,423	84.1%	14,595	15.9%	92,018	100.0%
Cholesterol														
# Screened	23,697	38.1%	11,419	38.2%	3,799	24.5%	31,317	40.9%	28,103	36.3%	7,013	48.1%	35,116	38.2%
# Referred to primary care	3,620	15.3%	1,612	14.1%	275	7.2%	4,957	15.8%	4,345	15.5%	887	12.6%	5,232	14.9%
Blood pressure														
# Screened	60,877	98.0%	29,335	98.1%	15,040	97.0%	75,192	98.3%	75,970	98.1%	14,262	97.7%	90,212	98.0%
# First time identified deviations from the norm	4,287	7.0%	2,641	9.0%	834	5.5%	6,094	8.1%	5,774	7.6%	1,154	8.1%	6,928	7.7%
# Treatment monitoring*	15,577	25.6%	6,329	21.6%	491	3.3%	21,415	28.5%	19,285	25.4%	2,621	18.4%	21,906	24.3%
Diabetes														
# Screened	45,809	73.7%	21,804	72.9%	10,335	66.6%	57,278	74.9%	57,081	73.7%	10,532	72.2%	67,613	73.5%
# First time identified deviations from the norm	3,560	7.8%	1,512	6.9%	386	3.7%	4,686	8.2%	3,864	6.8%	1,208	11.5%	5,072	7.5%
# Treatment monitoring*	3,470	7.6%	1,443	6.6%	161	1.6%	4,752	8.3%	4,292	7.5%	621	5.9%	4,913	7.3%
Cancer screening														
Colorectal cancer														
# Screened	38,276	61.6%	25,173	84.2%	8,861	57.1%	54,588	71.4%	55,315	71.4%	8,134	55.7%	63,449	69.0%
# Referred to a doctor/PHC	6,119	16.0%	4,741	18.8%	527	5.9%	10,333	18.9%	9,238	17%	1,622	20%	10,860	17.1%
Prostate cancer - for male only														
# Screened	n/a	n/a	25,694	85.9%	3,934	75.2%	21,760	88.2%	23,418	88.7%	2,276	64.9%	25,694	85.9%
# Referred to a doctor/PHC	n/a	n/a	5,955	23.2%	320	3.6%	5,635	25.9%	5,389	23%	566	25%	5,955	23.2%
Breast cancer - for female only														
#Screened	51,828	83.4%	n/a	n/a	8,407	81.8%	43,421	83.8%	44,861	87.9%	6,967	62.8%	51,828	83.4%
#Referred to a doctor/PHC	11,319	21.8%	n/a	n/a	1,256	14.9%	10,063	23.2%	9,728	22%	1,591	23%	11,319	21.8%
Cervical cancer - for female only														
# Screened	50,728	81.7%	n/a	n/a	8,334	81.1%	42,394	81.8%	43,829	85.9%	6,899	62.2%	50,728	81.7%
# Referred to a doctor/PHC	12,222	24.1%	n/a	n/a	1,616	19.4%	10,606	25.0%	10,566	24%	1,656	24%	12,222	24.1%
Mental health assessments														
screening using the PHQ9 questionnaire	53,935	86.8%	26,389	88.3%	14,008	90.3%	66,316	86.7%	69,012	89.1%	11,312	77.5%	80,324	87.3%
referral to a family doctor	3,972	7.4%	1,802	6.8%	894	6.4%	4,880	7.4%	4,755	6.9%	1,019	9.0%	5,774	7.2%
Conducting education session on stress management	29,680	55.0%	14,214	53.9%	7,079	50.5%	36,815	55.5%	36,994	53.6%	6,900	61.0%	43,894	54.6%
Immunization-related outreach														
screening	51,841	83.5%	25,168	84.2%	13,258	85.5%	63,751	83.3%	64,441	83.2%	12,568	86.1%	77,009	83.7%
referral to a doctor	7,656	14.8%	3,941	15.7%	1,887	14.2%	9,710	15.2%	9,451	14.7%	2,146	17.1%	11,597	15.1%

*A person is already registered and receiving treatment.

Screening rates for most NCDs were similar across age groups, with BP screening highest at 97.0% for those under 40 and 98.3% for those 40 and over. Mental health screenings were performed on 90.3% of individuals under 40 years old and 86.7% of those over 40. Breast and cervical cancer screening rates were high for women both under and over 40 at 81.8 and 83.8%, respectively. Screening rates for cholesterol and colorectal cancer were lower, with 24.5% vs. 40.9% for cholesterol and 51.7% vs. 71.4% for colorectal cancer in those under and over 40, respectively (*p* < 0.001).

Referral rates for values deviating from the norm (see Tables 2, 4) in those aged 18–39 years were 7.2% for cholesterol, 5.5% for blood pressure, 3.7% for diabetes, 5.9% for colorectal cancer, and 3.6% for prostate cancer. Referrals were 14.9% for breast cancer, 19.4% for cervical cancer, and 14.2% for needing a Td booster vaccination. Among those aged 18–39 years, 6.4% were referred to a family doctor after mental health assessment. NCD referrals were lower in this group compared to individuals aged 40 and above.

Cholesterol screening coverage was higher at community venues (48.1%) compared to medical venues (36.3%). Screening rates for blood pressure and diabetes were comparable between medical and community venues, with blood pressure screening conducted at 98.1% of medical sites and 97.7% of community venues, and diabetes screening performed at 73.7 and 72.2% of these sites, respectively. Cancer screening was substantially lower at community venues (55 to 65%) compared to medical sites (70 to 89%). Similarly, fewer attendees had their mental health assessed at community venues (77.5%) compared to 89.1% at medical sites. Cancer risk assessments resulted in referrals for 21.8% (breast), 24.1% (cervical), 23.2% (prostate), and 17.1% (colorectal) of screened individuals. Immunization checks revealed 15.1% of attendees required a Td booster.

## 4 Discussion

The establishment of HAPs in Ukraine demonstrated that community-embedded screening and referral can be implemented at scale under crisis conditions. Similar screening interventions have been implemented in other humanitarian and conflict-affected settings (3, 4). These interventions, like the HAPs in our setting, expanded access beyond facility-based services during crisis and brought screening and referral closer to communities, highlighting the potential of decentralization and community embedding to mitigate barriers in war-torn contexts. In Ukraine, setting up these sites supported continuity of preventive services when routine access to primary health care was constrained by insecurity, transport limitations, infrastructure damage, and workforce shortages. These barriers have been documented as key drivers of reduced health care access during the war, particularly in remote areas and among vulnerable populations (5). Evidence from Europe, including Ukraine, has shown persistent gaps in BP and lipid target achievement even among individuals engaged in PHC, indicating that prevention requires additional entry points and reinforcement beyond routine clinical encounters (18). A key innovation was expanding screening beyond the nationally mandated threshold of 40 years to include adults aged 18–39 years. Although most attendees were older, uptake among adults aged 18–39 years increased over time, and screening detected deviations from normal values in this younger age group, including elevated blood pressure and abnormal glucose and lipid measurements, as well as positive depression screens. This indicates that limiting screening to those aged 40 years and above may miss earlier risk and opportunities for timely referral.

HAP attendance patterns over time likely reflected changes in service availability and implementation dynamics rather than true seasonality in seeking health care. These fluctuations were primarily associated with phased expansion of HAPs, operational factors, and contextual influences such as seasonal agricultural activities and population movement during the war. Peaks coincided with expansion phases and reporting cycles, while declines—particularly in summer and early autumn—may also reflect rural livelihood patterns such as agricultural workload, given that many screening points were located in remote rural areas. Overall, fluctuations are best interpreted as operational and contextual effects rather than any reduced need for screening.

Placing HAPs in both medical and community venues offered complementary advantages. Medical sites enabled broader service delivery due to clinical staffing, while community venues expanded reach in settlements without functioning medical infrastructure and reduced psychological barriers among individuals reluctant to attend formal health facilities. Communities adapted the model based on local capacity, including the availability of community activists and the willingness of local authorities to support operations, which influenced both the number of operational sites and their performance. Strengthening interoperable referral tracking systems, including integration with national medical information systems, will be critical to enable monitoring of referral completion and patient outcomes.

A key limitation of this study is the absence of systematic data on referral completion, diagnostic confirmation, and treatment initiation. As a result, the effectiveness of the referral pathway and downstream health outcomes could not be assessed. The findings may be subject to selection bias, as individuals able to access HAP locations may differ from those facing the greatest barriers to care, including highly mobile, isolated, or insecure populations.

Implementation required ongoing supervision and adaptation. Infrastructure limitations, unstable electricity supply, delayed equipment delivery, and staff turnover necessitated repeated training and mentorship, particularly for mental health screening and cancer risk assessment (17). Local leadership played a central role in mobilizing participation, and hromadas with stronger engagement appeared to achieve better uptake and referral follow-through, reinforcing the importance of local ownership for sustainability. To ensure long-term resilience, the Ministry of Health should consider formalizing community-embedded screening as a recognized entry point to the PHC system, particularly for de-occupied and rural hromadas. Moving forward, the integration of the Medical information system (MIS), a common weakness of decentralized screening models, was addressed by linking screening results at the HAPs directly to the PHC digital systems, allowing PHC doctors to access data in real time thus strengthening continuity of care. This systems approach is consistent with Ukraine’s broader direction toward public health system strengthening and more systematic prevention delivery (5).

The HAP model aligns with and complements other Ukrainian initiatives addressing NCD screening gaps in war-affected areas. For instance, mobile health units (MHUs) deployed by the Ukrainian Red Cross and WHO deliver essential primary care to IDPs and hard-to-reach communities, focusing on strengthening local systems amid hostilities. Similarly, Pact’s Public Health System Strengthening Collaborative, launched in 2024, employs nurses and mobile teams to achieve 60–98% NCD screening coverage in rural hromadas, surveying over 25,000 residents and identifying risks in 80%. The national “Health Screening 40+” program, operational since January 2026, invites adults 40 + for free check-ups at registered facilities, emphasizing cardiovascular, diabetes, and mental health screening.

Unlike vehicle-based MHUs, HAPs leverage fixed community-embedded venues (e.g., libraries, social centers) for sustained accessibility without mobility logistics, enabling broader scale (234 sites) and inclusion of younger adults (18–39 years). This stationary, decentralized approach fills gaps in facility-based programs like Health Screening 40 + by generating demand and referrals in de-occupied and IDP-heavy hromadas, promoting integration across PHC entry points. These synergies underscore HAPs’ role in Ukraine’s evolving preventive framework during ongoing conflict.

## 5 Conclusion

The Health Access Point model proved to be a feasible and scalable extension of the Ukrainian primary healthcare system during a period of extreme systemic stress, when routine access to PHC was constrained by insecurity, transport limitations, infrastructure damage, and workforce shortages. By utilizing existing community infrastructure and non-medical personnel, the project helped to expand access to preventive services and reach additional individuals with often “hidden” health risks—particularly those with limited access to health care—including elevated blood pressure and unmet mental health needs requiring further assessment. Importantly, the model was designed to complement—not replace—facility-based PHC. HAP activities focused on national screening protocols, while diagnostic confirmation, clinical decision-making, and treatment initiation remained within PHC and specialist services. The model’s added value included flexible delivery through existing medical sites and certain community venues (e.g., libraries), strengthened community awareness and demand for screening, and broadening of the target group for screening to those aged below 40 years. Future research should include longitudinal tracking of referral completion and clinical outcomes to evaluate the effectiveness of the care pathway initiated through HAPs.

During the project period (December 2023 to June 2025), Ukraine further strengthened the national framework for preventive screening and early detection (including updated ministerial orders and government decisions on state-guaranteed screening services). Building on these policy developments, the STBCEU project is closely collaborating with the Ukrainian Center for Public Health and other national partners to advance implementation, address remaining access gaps, and align community-embedded approaches with the evolving national system. Future efforts should prioritize hromadas with limited facility access and high vulnerability, strengthen interoperable referral tracking to improve follow-up, and maintain continuous training, supervision, and reliable logistics to normalize preventive screening as a routine PHC-linked service—especially in fragile and disrupted settings.

## Data Availability

The raw data supporting the conclusions of this article will be made available by the authors, without undue reservation.
